# A multi-center inter-manufacturer study of the temporal stability of phase-contrast velocity mapping background offset errors

**DOI:** 10.1186/1532-429X-14-S1-W48

**Published:** 2012-02-01

**Authors:** Peter D Gatehouse, Marijn P Rolf, Karin Markenroth Bloch, Philip J Kilner, Martin J Graves, David N Firmin, Mark B Hofman

**Affiliations:** 1 Royal Brompton Hospital, London, UK; 2 VU University Medical Center, Amsterdam, Netherlands; 3 University of Lund, Lund, Sweden; 4 Addenbrookes Hospital, Cambridge, UK

## Summary

In the absence of previously published data, this work aimed to assess the temporal stability of the background offset.

## Background

Phase-contrast velocity images often contain a background or baseline offset error, adding an unknown offset to the measured velocities. For accurate flow measurements, this offset must be corrected or shown to be negligible. Some correction techniques depend on replicating the clinical flow acquisition using a uniform stationary phantom, in order to measure the baseline offset at the region of interest and subtract it from the clinical study. Such techniques assume that the background offset is stable over the time of a patient scan, or longer if the phantom scans are acquired later, or if the corrections are derived from pre-stored background correction images. Factors such as gradient power amplifier heating, and variations in eddy currents or their corrections are liable to influence stability. In the absence of previously published data, this work aimed to assess the temporal stability of the background offset.

## Methods

A fixed phase-contrast retro-gated cine acquisition at a fixed location and orientation was repeated 5 times in rapid succession, and this was performed weekly on stationary uniform phantoms using 3 different manufacturers’ scanners for 8 weeks. The largest change in the background offset within 50mm of isocenter was determined, defining a change of >=0.6cm/s as significant. The 8 week interval did not include any relevant system service visits. Any automatic background correction available was disabled since its performance in uniform phantoms is irrelevant.

## Results

**Figure 1:**
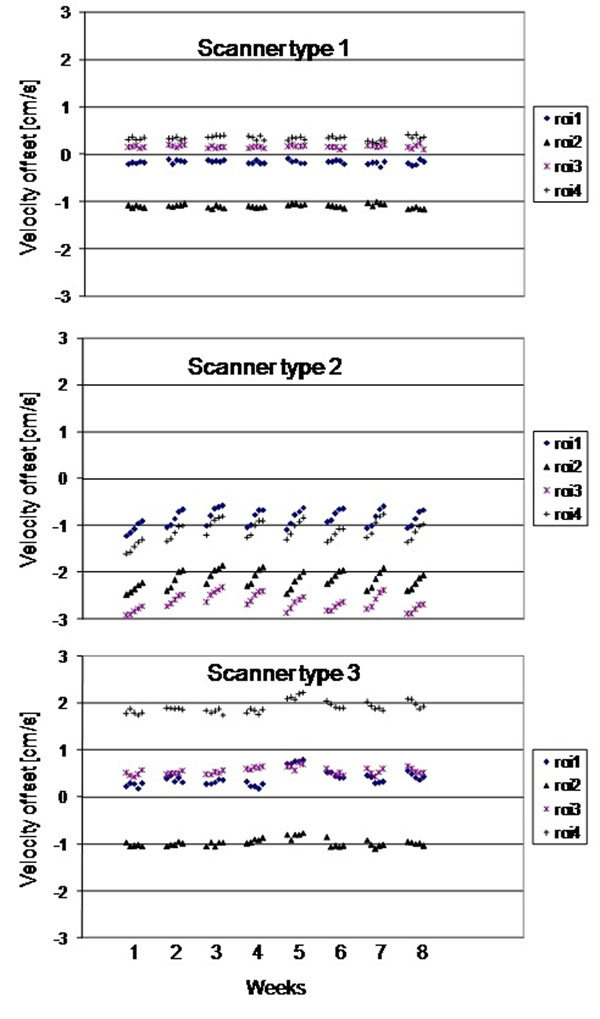
For the 3 types of scanned, the background offset errors are shown at four ROIs positioned 50mm from isocenter, showing each of the 5 scans in each weekly session, for weeks 1-8.

**Figure 2:**
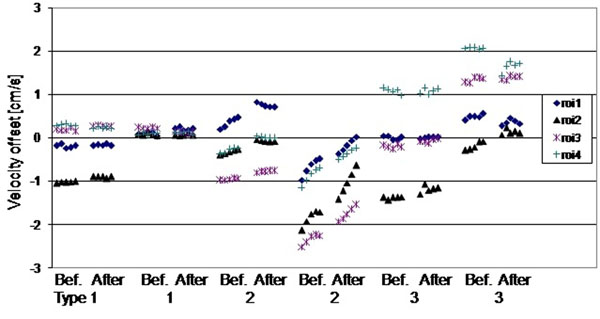
The ROI mean values from 2 scanners of each type, before and after high-power scanning. The horizontal gaps between the groups of five scans before and after high-power scanning are not to temporal scale, they were approximately as long as 20 velocity cines.

Over the several minutes timescale of the 5 repeats (Figure 1), insignificant temporal drift (0.1cm/s, 0.2cm/s) in the baseline offset was found on scanner types 1 and 3, with a marginally insignificant 0.5cm/s on type 2, caused by an apparent short-term heating effect. This finding was investigated with additional tests, furthermore replicated in a second identical set of the same 3 scanner types, in which the difference caused after ≈ 5 minutes of high gradient duty-cycle scanning was measured (0.1cm/s, 0.8cm/s, 0.4cm/s worst-case for types 1,2,3 with variation in amount of drift between machines of the same type, Figure 2). Over a longer timescale of 8 weeks (Figure 1), insignificant drift (0.2 cm/s) occurred on scanner type 1, with larger drifts (0.9cm/s, 0.6cm/s) on types 2 and 3.

## Conclusions

Over the duration and activity of a typical patient study, background drift was insignificant. However, the combination of extended high gradient power scanning with work requiring background correction requires care to avoid drift on some machines. Over the longer term of 8 weeks, significant drift is likely, preventing accurate correction by delayed phantom correction scans or derivation from pre-stored background offset images.

## Funding

NIHR Cardiovascular Biomedical Research Unit funding.

